# HF-SENSE: an improved partially parallel imaging using a high-pass filter

**DOI:** 10.1186/s12880-019-0327-3

**Published:** 2019-04-03

**Authors:** Jucheng Zhang, Yonghua Chu, Wenhong Ding, Liyi Kang, Ling Xia, Sanjay Jaiswal, Zhikang Wang, Zhifeng Chen

**Affiliations:** 1 Department of Clinical Engineering, 2nd Affiliated Hospital, School of Medicine, Zhejiang University, Hangzhou, Zhejiang China; 2 Department of Radiology, 2nd Affiliated Hospital, School of Medicine, Zhejiang University, Hangzhou, Zhejiang China; 3 Department of Biomedical Engineering, Zhejiang University, Hangzhou, Zhejiang China; 4 State Key Lab of CAD & CG, Zhejiang University, Hangzhou, Zhejiang China; 5 School of Medicine, Zhejiang University, Hangzhou, Zhejiang China; 6 School of Biomedical Engineering, Guangdong Provincial Key Laboratory of Medical Image Processing, Southern Medical University, Guangzhou, China

**Keywords:** SENSE, Image reconstruction, High pass filter, Artificial sparsity

## Abstract

**Background:**

One of the major limitations of MRI is its slow acquisition speed. To accelerate data acquisition, partially parallel imaging (PPI) methods have been widely used in clinical applications such as sensitivity encoding (SENSE) and generalized autocalibrating partially parallel acquisitions (GRAPPA). SENSE is a popular image-domain partially parallel imaging method, which suffers from residual aliasing artifacts when the reduction factor goes higher. Undersampling the k-space data and then reconstruct images with artificial sparsity is an efficient way to accelerate data acquisition. By exploiting artificial sparsity with a high-pass filter, an improved SENSE method is proposed in this work, termed high-pass filtered SENSE (HF-SENSE).

**Methods:**

First, a high-pass filter was applied to the raw k-space data, the result of which was used as the inputs of sensitivity estimation and undersampling process. Second, the adaptive array coil combination method was adopted to calculate sensitivity maps on a block-by-block basis. Third, Tikhonov regularized SENSE was then used to reconstruct magnetic resonance images. Fourth, the reconstructed images were transformed into k-space data, which was filtered with the corresponding inverse filter.

**Results:**

Both simulation and in vivo experiments demonstrate that HF-SENSE method significantly reduces noise level of the reconstructed images compared with SENSE. Furthermore, it is found that HF-SENSE can achieve lower normalized root-mean-square error value than SENSE.

**Conclusions:**

The proposed method explores artificial sparsity with a high-pass filter. Experiments demonstrate that the proposed HF-SENSE method can improve the image quality of SENSE reconstruction. The high-pass filter parameters can be predefined. With this image reconstruction method, high acceleration factors can be achieved, which will improve the clinical applicability of SENSE.

This retrospective study (HF-SENSE: an improved partially parallel imaging using a high-pass filter) was approved by Institute Review Board of 2nd Affiliated Hospital of Zhejiang University (ethical approval number 2018–314). Participant for all images have informed consent that he knew the risks and agreed to participate in the research.

## Background

Magnetic Resonance Imaging (MRI) is an important technology in modern medical imaging. It is a routine clinical examination method which provides superior soft-tissue characterization with flexible image contrast parameters [1]. One of the major limitations of MRI is its slow acquisition speed [2]. To accelerate image acquisition, partially parallel imaging (PPI) methods have been widely used in clinical applications such as sensitivity encoding (SENSE) and generalized autocalibrating partially parallel acquisitions (GRAPPA) [3, 4]. In addition, PPI methods can reduce image blurring and distortion from accelerated echo planar imaging (EPI) data [5]. SENSE is the most widely used image-domain based PPI technique, which requires coil sensitivity information to eliminate the effect of undersampling in the k-space [3, 6]. As the reduction factor *R* increases, errors exist in the coil sensitivity estimation lead to higher noise level and more residual aliasing artifacts. In theory, the signal-to-noise ratio (SNR) of the SENSE reconstructed image is reduced by $\sqrt{R}$ due to reduced Fourier averaging [1].

According to Storey et al., the noise amplification would be lower if the image content is sparse, because fewer nonzero pixels are superimposed in the aliased images [7]. This has also been proved by Blaimer et al. in ISMRM 2008 [8]. Over the last few years, it is found that PPI performed better on to-be-restored images with smaller image support, such as the observation in *k-t* GRAPPA [9] and other works [10–16]. The phrase “image support” can be defined as the “nonzero” regions in an image. Refer to hp-GRAPPA [12], image support reduction can be descriptively defined as the reduction of “nonzero” regions in an image, which mainly consist of low-frequency information. It is well known that the low-frequency information of an image is located at the center of its k-space. Hence, suppressing the central k-space data will reduce the image support. The low SNR and high g-factor caused by high reduction factor can be improved by artificial sparsity [14–16]. It should be noted that the phrase “artificial sparsity” is different from the popular “sparse MRI”, which is related to compressed sensing [17–19]. About artificial sparsity, Huang, et al. proposed a method named hp-GRAPPA, which used a high-pass filter *f* to efficiently reduce the image support [12]. The high-pass filter *f* and its inverse *1/f* are applied before and after GRAPPA reconstruction respectively, which significantly reduces the artifact level in the final reconstructed image. This method was verified with simulation data, in vivo cardiac and brain data experiments. Chen, et al. proposed an artificial sparsity-based method for non-Cartesian trajectory, both simulation and in vivo imaging experiments demonstrated that this approach can effectively improve the SNR and reduce the g-factor values [14]. They further extended this work to non-Cartesian dynamic contrast-enhanced MRI by exploiting dynamic artificial sparsity [15]. Wang, et al. also investigated artificial sparsity in dynamic MRI using an improved *k-t* principal component analysis algorithm with different reduction factors [16].

So far, the above-mentioned artificial sparsity methods are mostly based on GRAPPA-type algorithms. A SENSE-like framework known as skipped phase encoding and edge deghosting with array coil enhancement (SPEED-ACE) applied a high-pass filter to the ghosted images corresponding to undersampling, resulting in a set of sparse ghosted edge maps, which were combined into a deghosted edge map [20]. The deghosting process was implemented through least-squares-error method on a pixel-by-pixel basis, thus the SPEED-ACE method exploit artificial sparsity in image space.

In this research, we have proposed an MRI reconstruction technique, which combines SENSE with artificial sparsity, termed high-pass filtered SENSE (HF-SENSE). The high-pass filter *f* and its inverse *1/f* are applied before and after SENSE reconstruction respectively. Its effectiveness is validated by using both numerical simulation and in vivo datasets with different coil numbers and different pulse sequences.

## Methods

### Brief review of SENSE

SENSE is an SNR-optimal reconstruction approach along with a coil sensitivity map that ensures the accuracy of the restoring procedure [3, 21]. According to Hoge et al., SENSE is a combine-then-reconstruct method, the accuracy of SENSE reconstruction mainly depends on the accuracy of coil sensitivity information [22]. The completed pipeline is explained in the following using a specific instance.

**Fig. 1:**
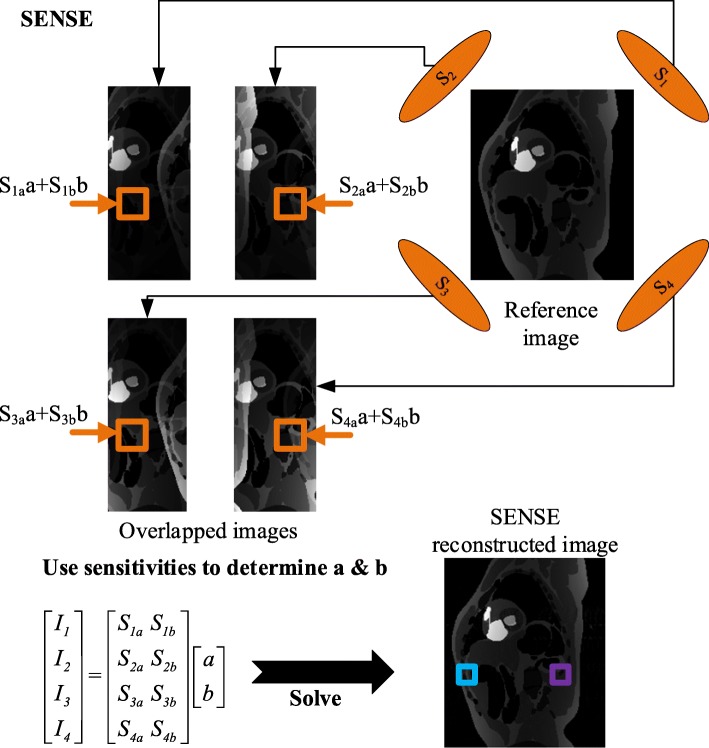
The basic flowchart of SENSE

Consider a case of 4 receiver channels, and the reduction factor is set to 2, as shown in Fig. 1. First of all, the folded images are obtained from subsampled k-space data coil-by-coil through Fourier transform operators. And then, use the coil sensitivity information *S* to unfold the aliased image. This procedure is implemented through the concatenation of folded matrix, as displayed in Fig. 1.

In the reduced FOV, assume *I*_*A*_ is the pixel value of aliased image, *S* represents the coil sensitivity map, *I*_*o*_ is un-aliased image, *n* represents for noise. *I*_*A*_ can be written as the following:

1 $$ {I}_A=S\cdot {I}_o+n $$

Suppose the expectation of *n* is 0, the covariance is Ψ, i.e., Ψ = cov(*n*), hence the un-aliased image can be represented as:

2 $$ {I}_o={\left({S}^H\Psi S\right)}^{-1}{S}^H\Psi {I}_A $$

Here *H* is the Hermite transpose, represents the conjugate transpose of complex number. Assume that *U* = (*S*^*H*^Ψ*S*)^−1^*S*^*H*^Ψ, and *U* is called the unfolding matrix. Then the former equation can be rewritten as:

3 $$ {I}_o={UI}_A $$

SENSE is appropriate for almost all kinds of phased array coils, and it is a flexible technique which is widely used in many clinical applications [3, 23, 24]. However, it suffers from noise amplification and residual aliasing artifacts when the accelerating factor goes higher [3, 21]. Tikhonov regularization is used to solve the ill-conditioned linear equations of Eq. (1):

4 $$ {I}^{\lambda }=\underset{I}{\arg \min}\left\{{\left\Vert S\cdot I-{I}_A\right\Vert}_2+{\lambda}^2{\left\Vert \left(I-{I}_1\right)\right\Vert}_2\right\} $$

where *λ*^2^ is the regularization factor, *I*_1_ denotes the prior information about the solution *I*, and ‖•‖_2_ represents the L-2 norm. The regularization factor *λ*^2^ quantifies the trade-off between the error from noise amplification from the unconditioned matrix inversion, and the error from prior knowledge not describing the current image [25, 26].

### High-pass filter

**Fig. 2:**
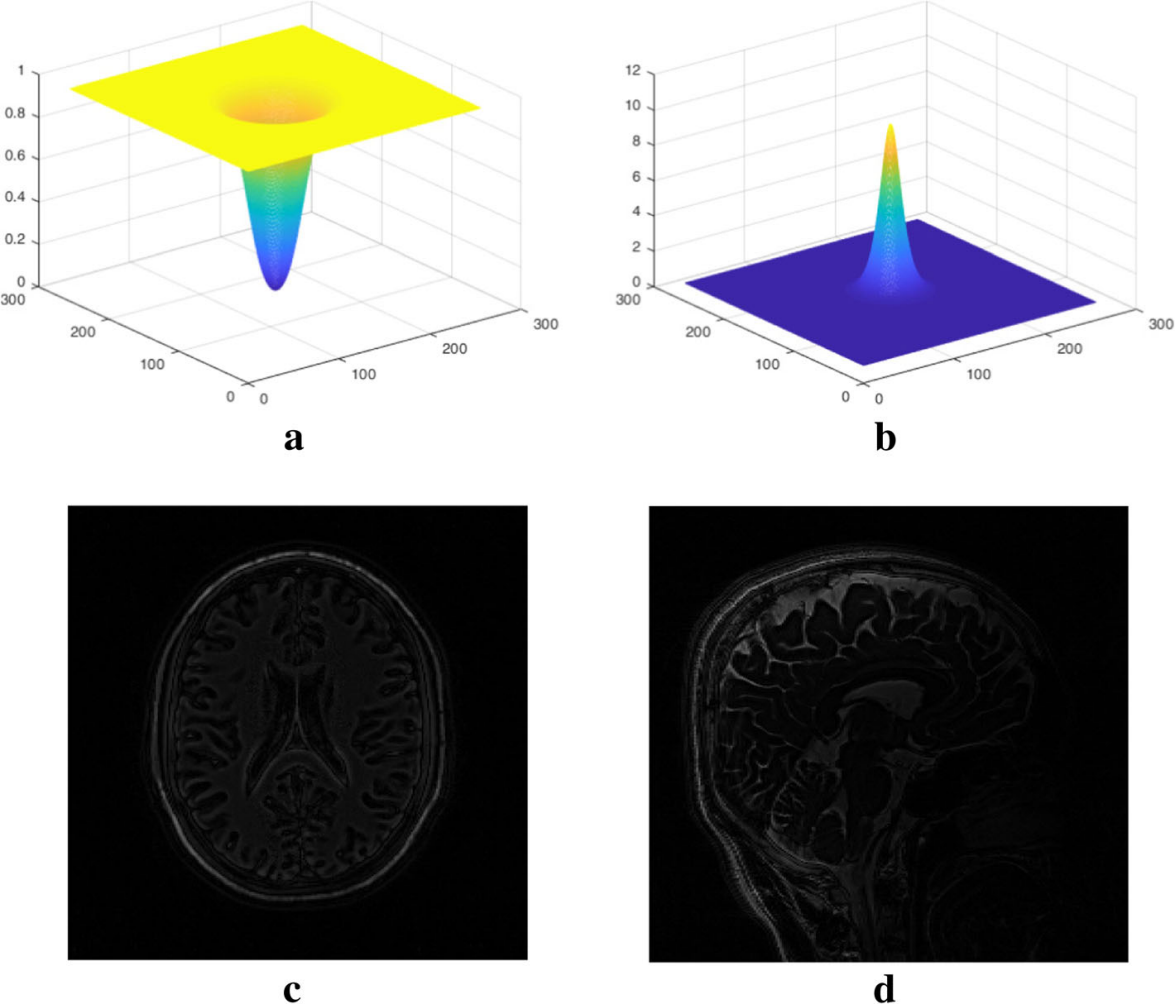
**a** High-pass filter and **b** its inverse filter (*c* = 24 and *w* = 8); **c** simulated axial brain image after filtering, and **d** sagittal brain image after filtering

The same high-pass filter as Filter 2 in hp-GRAPPA is adopted in this process [12]:

5 $$ F=1-{\left(1+{e}^{\left(\sqrt{k_x^2+{k}_y^2}-c\right)/w}\right)}^{-1}+{\left(1+{e}^{\left(\sqrt{k_x^2+{k}_y^2}+c\right)/w}\right)}^{-1} $$

where *k*_*y*_ is the count of phase encoding lines, *k*_*x*_ is the count of frequency encoding lines, *c* sets the cutoff frequency and *w* determines the smoothness of the filter boundary. An example of the high-pass filter and its corresponding inverse filter while *c* = 24 and *w* = 8 were shown in Fig. 2. It was found that larger *c* or smaller *w* suppresses the image support more, these parameters can be predefined in the experiment.

### HF-SENSE

**Fig. 3:**
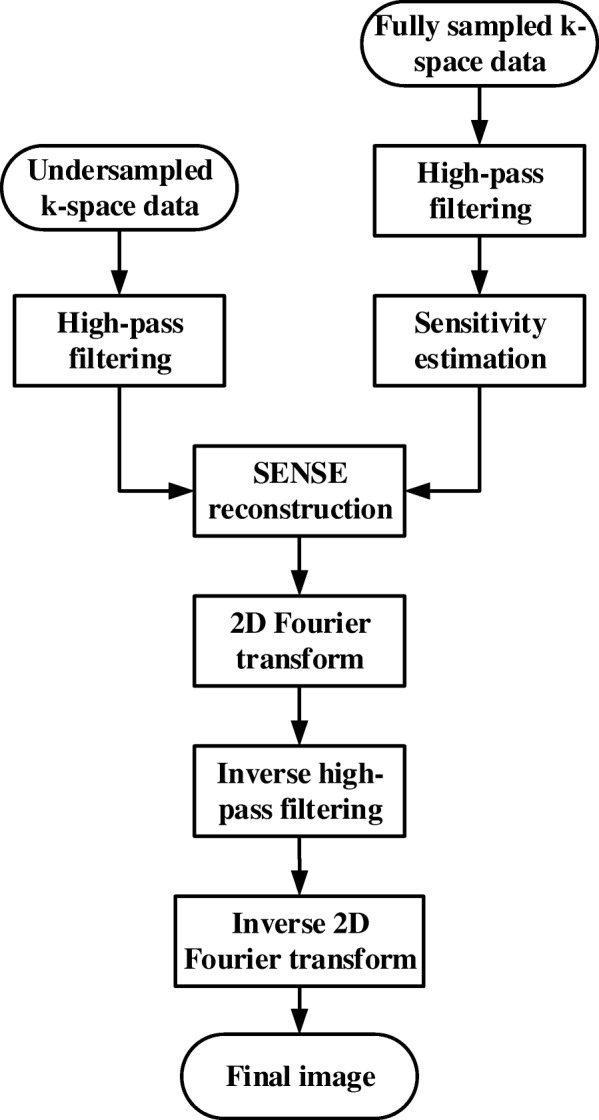
The flowchart of HF-SENSE. The k-space raw data is high-passed before undersampling and sensitivity estimation. The SENSE reconstructed image is transformed into k-space data, which is filtered with the corresponding inverse high-pass filter. Final image is obtained by applying inverse 2D Fourier transform to this k-space data

Figure 3 depicts the flowchart of the proposed HF-SENSE method. Instead of the full k-space data, the high-pass filtered k-space data is used to calculate coil sensitivity information with Walsh’s adaptive array-combination method [27, 28]. The signal array correlation matrices and matched filter vector were computed on a block-by-block basis. A unique filter vector was computed and applied to each 4 × 4 block of pixels, using an 8 × 8 pixel estimation region for the local signal statistics. The noise correlation matrix was assumed to be the identity matrix. After undersampling the k-space data, Tikhonov regularized SENSE was used to reconstruct the image. To apply the corresponding inverse filter, the SENSE reconstructed image was transformed to k-space with two-dimensional (2D) Fourier transform. Final HF-SENSE reconstructed image was obtained with the corresponding inverse 2D Fourier transform.

It is widely adopted that partially parallel imaging has good performance when the to-be-restored image is sparse [7–16]. For SENSE-like approach, the noise amplification would be lower if the image content is sparse, because fewer nonzero pixels are superimposed in the aliased images [7, 8]. The condition of SENSE equation will be improved when artificial sparsity is included. For GRAPPA-like method, this kind of parallel imaging will consider image content inherently [7]. The proposed HF-SENSE uses a high-pass filter to select the high-frequency part of k-space, corresponding to sparse image content.

### Experiment design

One simulated axial brain dataset and two in vivo datasets were investigated in this study. The simulated data was obtained from a noise-free 8-channel T1-weighted brain image. Gaussian noise was added to the simulation data. The acceleration factor of the simulated dataset was 4. One brain dataset was acquired on a 1.5 Tesla Area MR Scanner (Siemens AG, Erlangen, Germany) with a 10-channel head coil. The acquisition parameters of the T_2_ sequence included: field of view 230 × 230*mm*^2^, TR/TE = 3860/97 ms, oversampling ratio 2, slice thickness 6 mm, and flip angle 150°. The acceleration factor of the in vivo brain dataset was 4. Another in vivo knee imaging was performed on a 1.5 Tesla Avanto MR scanner (Siemens AG, Erlangen, Germany) using an 8-channel knee coil. The T_1_ sequence employed acquisition parameters: field of view 180 × 180*mm*^2^, TR/TE = 550/18 ms, oversampling ratio 2, slice thickness 4 mm, and flip angle 150°. The acceleration factor of the in vivo knee dataset was 4. All the datasets were reconstructed offline in the Matlab (R2017; MathWorks, Natick, MA) programming environment.

### Evaluation criteria

Using the reconstructed image with square root of sum-of-squares (SSOS) as reference, difference maps between images reconstructed with HF-SENSE (or SENSE) and SSOS were shown. Normalized root-mean-square error (NRMSE) was also calculated to evaluate the simulation and in vivo results. The NRMSE is defined as follow:

6 $$ NRMSE=\sqrt{\sum {\left({I}_{ref}(r)-I(r)\right)}^2/\sum {\left({I}_{ref}(r)\right)}^2} $$

where *I*_*ref*_(*r*) denotes the reference image; *I*(*r*) is the undersampled reconstruction result, and *r* is the spatial location of the image. The NRMSE is calculated within the whole image.

## Results

### Simulations

**Fig. 4:**
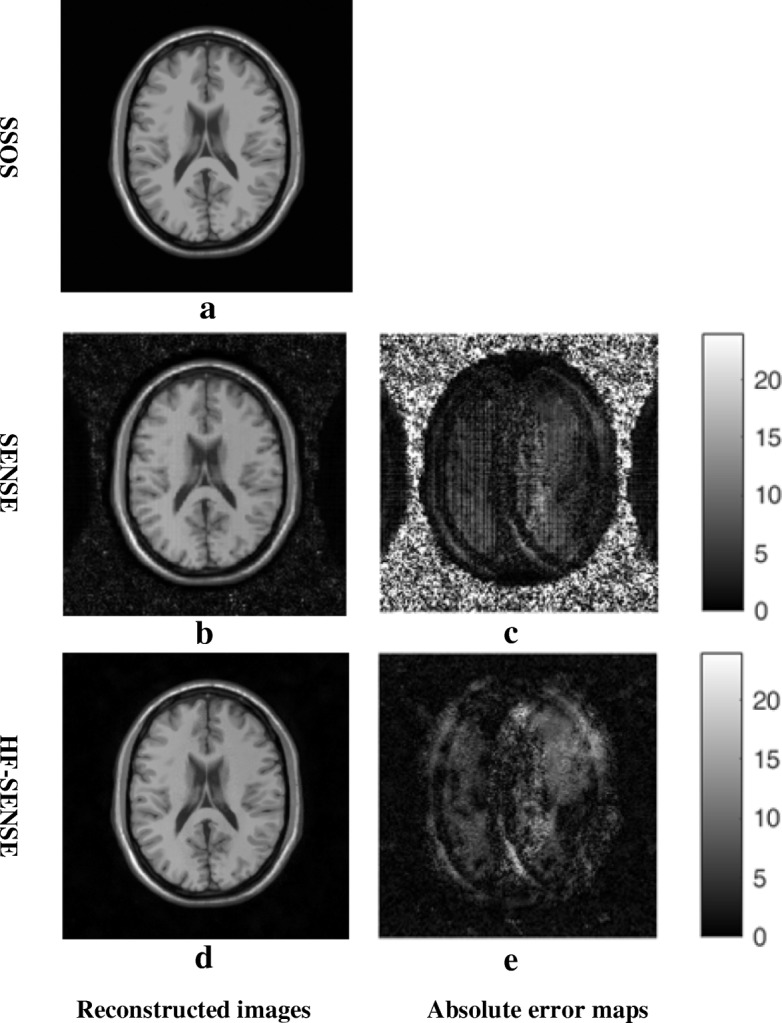
Results of the simulated axial brain dataset. *c* = 24 and *w* = 8 were used in the high-pass filter. The acceleration factor was 4. **a**, **b** and **d** the image reconstructed with SSOS, Tikhonov regularized SENSE (*λ* = 0.01) and HF-SENSE (*λ* = 0.001). **c** The absolute error map of (**a**) and (**b**). **e** The absolute error map of (**a**) and (**d**). **c** and **e** were brightened 10 times for better visualization

Figure 4a shows the results of simulated axial brain dataset. The acceleration factor was 4. The parameters of high-pass filter c and w were set to be 24 and 8, respectively. The left columns show the reconstructed images with SSOS, SENSE and HF-SENSE, which are scaled into the same color range. Taking the SSOS result as reference, the NRMSEs of HF-SENSE and SENSE reconstructed images with different regularization paramters are shown in Table 1. NRMSEs of HF-SENSE are lower than that of SENSE when the regularization factor is lower than 0.01. Figure 4b shows the SENSE reconstructed image while *λ* = 0.01 and Fig. 4d is the HF-SENSE reconstructed image while *λ* = 0.001. The SENSE reconstructed image is noisier than that of HF-SENSE (Fig. 4b Vs. d). This is more obvious considering the absolute error maps (Fig. 4c Vs. e).

**Table 1:** NRMSEs of SENSE and HF-SENSE reconstructed images with different regularization parameters

*λ*	NRMSE	
	SENSE	HF-SENSE
0.1	44.5%	45.5%
0.01	17.2%	5.4%
0.001	17.5%	5.2%
0.0001	17.9%	5.3%

### In vivo experiments

**Fig. 5:**
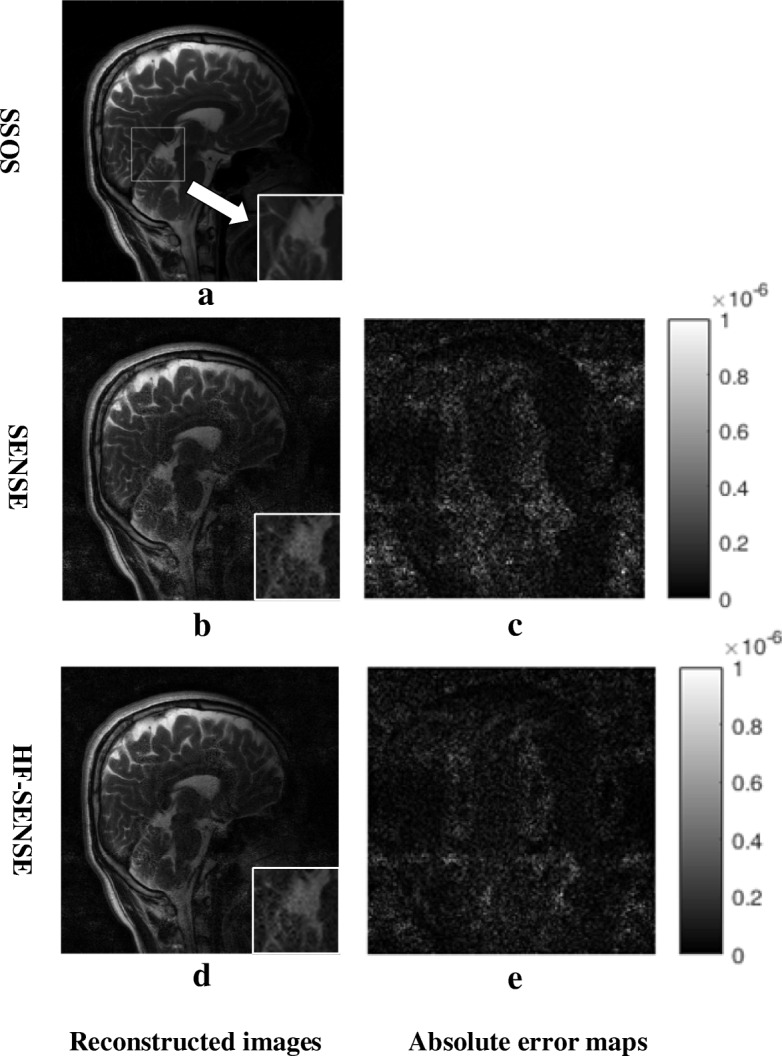
Results of the sagittal brain dataset. The Acceleration factor was 4. *c* = 24 and *w* = 12 were used in the high-pass filter. **a**, **b** and **d** the image reconstructed with SSOS, Tikhonov regularized SENSE (*λ* = 0.01) and HF-SENSE (*λ* = 0.01), the small figures in the right bottom corner of each figure depict the corresponding zoomed in region defined by the white box. **c** The absolute error map of (**a**) and (**b**). **e** The absolute error map of (**a**) and (**d**). **c** and **e** were brightened 4 times for better visibility

Figure 5 shows the results of the 5th sagittal slice acquired with a 10-channel brain coil. The left column shows the reconstructed images by SSOS, SENSE and HF-SENSE. The white square in Fig. 5a shows the location of a selected region of interest (ROI). The small figures in the right bottom corner of each figure demonstrate the corresponding zoomed in ROI defined by the white box. Taking the SSOS result as reference, the NRMSEs of HF-SENSE and SENSE reconstructed images with different regularization parameters are shown in Table 2. Figure 5b and d show the SENSE and HF-SENSE reconstructions with *λ* = 0.01 individually. It is observed that the SENSE reconstructed image is pretty noisy, and boundaries of cerebellum are seriously blurred. The noise of HF-SENSE reconstructed image (Fig. 5d) is greatly suppressed compared with SENSE reconstruction (Fig. 5b). As can be seen from the HF-SENSE reconstructed image and the corresponding enlarged image (Fig. 5d), the blurred edges of cerebellum are restored. Figure 5c shows the difference map between Fig. 5a, b, and e shows the difference map between Fig. 5a and d. The difference maps are scaled in the same range and brightened 4 times for better visibility. The difference maps (Fig. 5c Vs. e) further confirm that the noise level in the image reconstructed by HF-SENSE method is reduced compared with that of SENSE. Consequently, HF-SENSE shows better reconstructed image quality than SENSE.

**Table 2:** NRMSEs of SENSE and HF-SENSE reconstructed images with different regularization parameters

*λ*	NRMSE	
	SENSE	HF-SENSE
0.1	28.7%	26%
0.01	22%	18.2%
0.001	22%	18.2%
0.0001	22%	18.2%

**Fig. 6:**
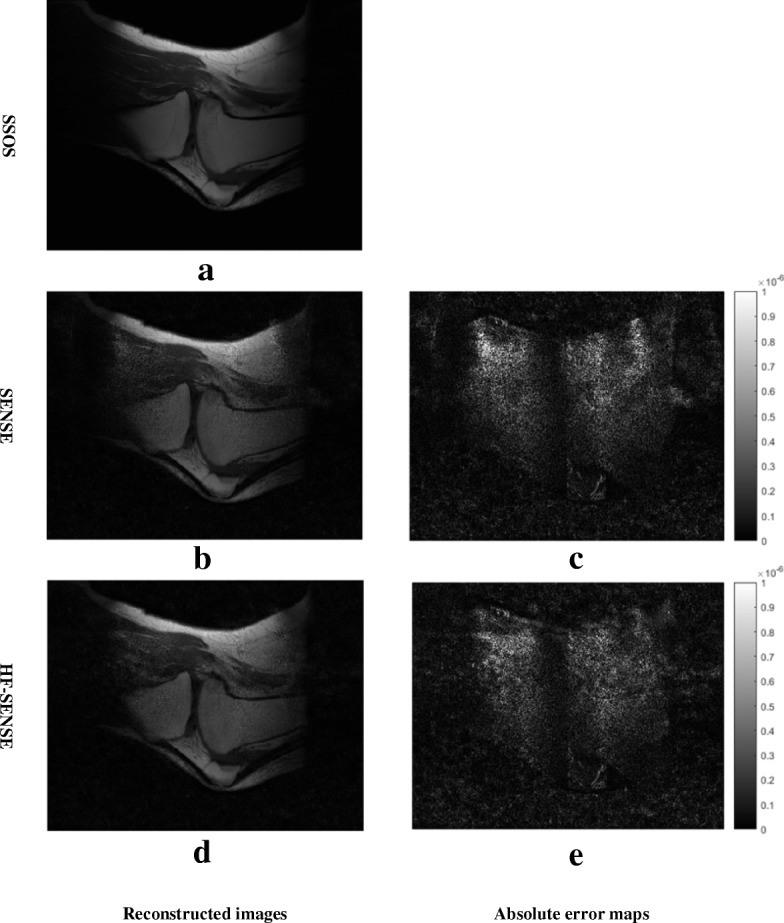
Results of the sagittal knee dataset. The acceleration factor was 4. **a**, **b** and **d** the image reconstructed with SSOS, Tikhonov regularized SENSE, and HF-SENSE. **c** The absolute error map of (**a**) and (**b**). **e** The difference map of (**a**) and (**d**). **c** and **e** were brightened 5 times for better visibility

Figure 6 shows the results of the 6th sagittal slice acquired with an 8-channel knee coil. The parameters of high-pass filter *c* and *w* were set to 24 and 8, which is the same as the simulation experiment. The left column shows the reconstructed images by SSOS, SENSE, and HF-SENSE. Taking the SSOS result as reference, the NRMSEs of HF-SENSE and SENSE reconstructed images with different regularization parameters are shown in Table 3. Figure 6b and d are the SENSE and HF-SENSE reconstructions while *λ* = 0.01, respectively. It is observed that lower noise level can be found on HF-SENSE reconstructed image (Fig. 6d) than that of SENSE (Fig. 6b). This is confirmed with difference maps (Fig. 6c Vs. e), which were brightened 5 times for better visibility. The NRMSEs of HF-SENSE and SENSE are 21.8 and 24.2%, respectively. Therefore, the HF-SENSE reconstructed image quality is better than that of SENSE. Further decreasing the regularization parameter cannot significantly improve the image quality.

**Table 3:** NRMSEs of SENSE and HF-SENSE reconstructed images with different regularization parameters

*λ*	NRMSE	
	SENSE	HF-SENSE
0.1	24.9%	48.6%
0.01	21.8%	24.2%
0.001	21.8%	24.2%
0.0001	21.8%	24.2%

## Discussion

An improved SENSE method, termed HF-SENSE is proposed in this article. The adaptive array-combination method is adopted to calculate sensitivity maps on a block-by-block basis, both in HF-SENSE and SENSE. In theory, compared to the SSOS method, the root-mean-square noise level in background image regions of adaptive reconstructed image is reduced by as much as $\sqrt{N}$, where *N* is the number of coil in the receiver array. However, when the reduction factor goes higher, the SENSE reconstructed image is pretty noisy in the whole image, and some structure details are blurred. For example, the in vivo experiment showed that boundaries of cerebellum were undistinguishable when the reduction factor was increased to 4 (Fig. 5b).

Both simulation and in vivo experiments were tested, using difference maps and NRMSE as evaluation criteria. In vivo experiments include 10-channel brain imaging and 8-channel knee imaging, the k-space raw data of which were off-loaded from different MRI scanners. Both simulation and in vivo experiments demonstrated that HF-SENSE reconstructed image showed lower noise level and lower NRMSE value than SENSE. As shown in the simulation results, the NRMSEs of HF-SENSE and SENSE are 5.2 and 17.2%, respectively. As for the in vivo experiments, the NRMSEs of HF-SENSE are also significantly lower than that of SENSE, regardless of imaging applications, number of coil channels or MRI pulse sequences. Hence better image quality can be achieved with HF-SENSE than SENSE, which is in consistence with hp-GRAPPA.

The HF-SENSE algorithm is easy to implement, only a high-pass filter and its corresponding inverse filter are applied before and after SENSE reconstruction individually, both work in k-space, which would not add too much calculation burden to SENSE. It is found that the parameters defining the high-pass filter may be predefined. The same high-pass filter parameters (*c* = 24 and *w* = 8) were used in simulation and in vivo knee experiments, both achieve satisfied results. This is because the number of coil channel is both equal to 8 in these two experiments. In contrast, the 10-channel brain in vivo MRI reconstruction experiment used different high-pass filter parameters (*c* = 24 and *w* = 12).

There are several limitations remain in the proposed approach. One limitation is the parameter sensitive problem. The HF-SENSE is sensitive to the high-pass filter parameters (*c* & *w* in this work). It is quite possible that no improvement would be shown if the inappropriate parameters are used. This limitation is also applied to high-pass GRAPPA technique. Another limitation of the HF-SENSE is the reduction factor issue. When the reduction factor goes higher, the HF-SENSE will not perform so well as in the case of low and moderate reduction factors.

In this article, it was found that HF-SENSE generates both less noise and lower NRMSE values than SENSE. The feasibility of using HF-SENSE on other clinical applications, dynamic MRI for example, needs further investigation. In addition, HF-SENSE can be combined with other fundamentally different acceleration methods to further improve the reconstruction quality, such as non-Cartesian k-space undersampling [29, 30] and compressed sensing [17–19].

## Conclusions

To the best of our knowledge, artificial sparsity with a high-pass filter can improve the image quality of SENSE reconstruction, which is validated with both simulation and in vivo datasets. The high-pass filter parameters can be predefined. The noise of the reconstructed image is significantly suppressed with the HF-SENSE method. This will improve the clinical applicability of SENSE.

## Abbreviations

GRAPPA: Generalized autocalibrating partially parallel acquisitions
HF-SENSE: High-pass filtered SENSE
MRI: Magnetic resonance imaging
NRMSE: Normalized root-mean-square error
PPI: Partially parallel imaging
SENSE: Sensitivity encoding
SNR: Signal-to-noise ratio
SPEED-ACE: Skipped phase encoding and edge deghosting with array coil enhancement
SSOS: Square root of sum-of-squares
